# Ultrahigh-Density Linkage Map for Cultivated Cucumber (*Cucumis sativus* L.) Using a Single-Nucleotide Polymorphism Genotyping Array

**DOI:** 10.1371/journal.pone.0124101

**Published:** 2015-04-13

**Authors:** Mor Rubinstein, Mark Katzenellenbogen, Ravit Eshed, Ada Rozen, Nurit Katzir, Marivi Colle, Luming Yang, Rebecca Grumet, Yiqun Weng, Amir Sherman, Ron Ophir

**Affiliations:** 1 Department of Fruit Trees Sciences, Institute of Plant Sciences, Agricultural Research Organization, Volcani Center, Rishon Lezion, Israel; 2 Department of Fruit Trees Sciences, Institute of Plant Sciences, Agricultural Research Organization, Newe-Yaar Center, Ramat Yishai, Israel; 3 The Robert H. Smith Institute of Plant Sciences and Genetics in Agriculture, Faculty of Agriculture, Food and Environment, The Hebrew University of Jerusalem, Rehovot, Israel; 4 Plant Breeding, Genetics, Genomics and Biotechnology, Department of Horticulture, Michigan State University, East Lansing, Michigan, United States of America; 5 Department of Horticulture, University of Wisconsin, Madison, Wisconsin, United States of America; 6 United States Department of Agriculture (USDA)-ARS, Vegetable Crops Research Unit, Madison, Wisconsin, United States of America; USDA-ARS-SRRC, UNITED STATES

## Abstract

Genotyping arrays are tools for high-throughput genotyping, which is beneficial in constructing saturated genetic maps and therefore high-resolution mapping of complex traits. Since the report of the first cucumber genome draft, genetic maps have been constructed mainly based on simple-sequence repeats (SSRs) or on combinations of SSRs and sequence-related amplified polymorphism (SRAP). In this study, we developed the first cucumber genotyping array consisting of 32,864 single-nucleotide polymorphisms (SNPs). These markers cover the cucumber genome with a median interval of ~2 Kb and have expected genotype calls in parents/F_1_ hybridizations as a training set. The training set was validated with Fluidigm technology and showed 96% concordance with the genotype calls in the parents/F_1_ hybridizations. Application of the genotyping array was illustrated by constructing a 598.7 cM genetic map based on a ‘9930’ × ‘Gy14’ recombinant inbred line (RIL) population comprised of 11,156 SNPs. Marker collinearity between the genetic map and reference genomes of the two parents was estimated at R^2^ = 0.97. We also used the array-derived genetic map to investigate chromosomal rearrangements, regional recombination rate, and specific regions with segregation distortions. Finally, 82% of the linkage-map bins were polymorphic in other cucumber variants, suggesting that the array can be applied for genotyping in other lines. The genotyping array presented here, together with the genotype calls of the parents/F_1_ hybridizations as a training set, should be a powerful tool in future studies with high-throughput cucumber genotyping. An ultrahigh-density linkage map constructed by this genotyping array on RIL population may be invaluable for assembly improvement, and for mapping important cucumber QTLs.

## Introduction

Linkage maps and association studies are becoming increasingly valuable for plant genetic research [1–4] and as a tool to facilitate efficient plant breeding [2]. Coupling a high-density linkage map with a high-throughput genotyping tool makes that linkage map useful for future studies. First-generation markers such as amplified fragment length polymorphisms (AFLPs), restriction-enzyme fragment length polymorphisms (RFLPs), and random amplified polymorphic DNA (RAPD) were used to create genetic maps of low resolution (5–10 cM) [5]. The second-generation markers were simple sequence repeats (SSRs) and single-nucleotide polymorphisms (SNPs), which were widespread over the genome [6] and therefore commonly used for denser maps. Next-generation sequencing (NGS) developments, such as 454-FLEX GS and Illumina HiSeq platforms, shortened the discovery period for both SNPs and SSRs, especially in non-model organisms. However, tools for massive parallel genotyping were more available for SNPs than for SSR markers. Before the emergence of NGS and specifically, genotyping by sequencing (GBS) platforms, the most commonly used high-throughput genotyping platforms were Affymetrix SNP arrays and Illumina SNP bead-chips [7,8].

In the last 5 years, the use of SNP arrays has been integrated into plant breeding studies and has leveraged marker-assisted selection [8]. SNP arrays have been developed for species such as corn [6], rice [9], spruce [10], grape [11], apple [12], and peach [13] based on genomic information, and for spruce [10] and lentil [14] based on SNPs discovered in the transcriptome. The number of SNPs that compose the arrays has increased dramatically, from a few hundred to tens of thousands, with the use of NGS for SNP discovery. In rice, for example, array sizes range from 384 to 1 million SNPs designed on Illumina and Affymetrix platforms. Those arrays appeared in 10 different designs and together created a large SNP reservoir [15]. The SNP-array design is, in practice, the choice of SNPs that compose the array. Designing a SNP array from the transcriptome often works very well for association studies, whereas SNPs that are derived from whole-genome sequences are targeted for linkage mapping and population genetics [10], as well as large-scale, e.g. genome-wide, association studies [16].

The cucumber (*Cucumis sativus* L., 2n = 2*x* = 14) genome was the first of the Cucurbitaceae family to be fully sequenced [17]. Moreover, its genome size is relatively small (367 Mbp) [18]. With the recent advances in technology and instrumentation for sequencing plant genomes, significant progress is being made in developing genetic and genomic resources for cucumber. The whole genomes of three cucumber lines have been sequenced: the Northern China type (Chinese Long) ‘9930’ [17], the North American pickling type ‘Gy14’ [19], and a European inbred line ‘B10’ [20]. The genomic information has been utilized to learn about inter-species synteny among *Cucumis* species [17,21,22], characterize recombination events and segregation distortion [23], and develop a large collection of SSR markers [24]. Using these markers, several SSR-based genetic maps have been constructed in cucumber. The map developed by Ren et al. [23] using a mapping population from a cross between cultivated cucumber (‘Gy14’) and wild cucumber (*C*. *sativus* var. *hardwickii*, PI 183967) has the highest marker density (995 SSR loci), but nearly one-third of the mapped loci are in clusters due to significant structural rearrangements between the cultivated and wild cucumber [23]. Another linkage map consists of 735 marker loci, developed with a ‘Gy14’ × ‘9930’ F_2_ mapping population [19]. By synthesizing marker information from mapping populations, two consensus cucumber genetic maps have also become available containing 1,369 [25] and 1,681 [26] SSR loci or genes, respectively.

While these linkage maps have greatly facilitated genetic mapping [26–28] and molecular tagging [26] in cucumber, their marker density is far from satisfactory for robust marker-based association studies. For example, cultivated cucumbers have an average LD (linkage disequilibrium) block of ~55 Kbp which means >6,000 markers may be needed for accurate genome-wide association studies to capture the genetic variation of interest [29]. In addition, the cultivated cucumber has a very narrow genetic base [30–32], making it difficult to develop a high-resolution genetic map using low-throughput markers such as SSRs. Therefore, in this study, our objective was to develop an ultrahigh-density SNP-based linkage map for cucumber using genotyping array. We describe the design of the first cucumber SNP array as a high-throughput tool for parallel genotyping and its application on a recombinant inbred line (RIL) population developed from a cross between two of the cultivars with sequenced genomes, Gy14 and 9930.

## Materials and Methods

### Plant material

A RIL mapping population was developed for linkage analysis and map construction from a cross between two cultivated inbred cucumber lines, ‘Gy14’ [19] and ‘9930’ [17], whose draft genome assemblies are available. A single F_1_ plant from ‘Gy14’ × ‘9930’ was self-pollinated to produce 150 F_7_ or F_8_ RILs that were used for the genetic mapping. The F_2_ population from the same cross was previously used to develop a SSR-based linkage map in cultivated cucumber [19]. Four additional cucumber inbred lines were used to evaluate the usefulness of this SNP array: 'G421', 'H19', 'WI 2757' and 'True Lemon' ('TL').

### DNA extraction, hybridization and labeling

Unexpanded young leaves were collected into 2.0-ml microcentrifuge tubes, lyophilized in a freeze-dryer, and ground to a fine powder. Genomic DNA (gDNA) was extracted using the CTAB method [33] and purified with phenol/chloroform.

Labeling of DNA pools and hybridization were performed at the Weizmann Institute’s DNA array unit following the Agilent CGH protocol for comparative genomic hybridization (http://www.agilent.com); the hybridization temperature was modified to 55°C.

### Probe design

Long reads yielded by 454-GS Flex Titanium sequencing of the 'Gy14' genome [19] and 100-bp shotgun reads obtained by Illumina HiSeq 2000 sequencing of the '9930' genome were mapped with the ‘mosaik’ program (http://bioinformatics.bc.edu/marthlab/wiki/index.php/Software) to the cucumber ‘9930’ draft genome version 1 (downloaded from http://www.icugi.org). Three criteria were used for selection among the 97,015 putative SNPs: genome coverage, SNP call, and SNP quality. The genome-wide determination was performed by running a 2-Kb window and selecting the best SNP within each window. The best SNP was defined as the SNP with the highest sequence read coverage of the minor allele and a base call quality of at least 25 (PHRED scale) or 57 (Illumina scale). Four probes were designed per SNP. Two probes, as replicates, for each allele, with one replicate probe designed to detect the forward and the other the reverse DNA strand. The probes were T_m_ optimized as follows [34]:

$$Tm\;=\;\frac{\Delta \text{H}*1000}{\Delta \text{S }+\;1.9872\text{*log}\left(\frac{\text{Ct}}{\text{x}}\right)}\;-\;273.15$$ (1)

where ΔH is the sum over all nucleotides’ ΔH and ΔS is sum over all nucleotides’ ΔS with respect to the values described in Santa Lucia et al. [35] and chosen for a single SNP within a window of 20–35 bp.

To avoid paralogous SNPs, i.e., SNPs that are the result of repetitive sequences in the same genome, we used only reads that were mapped uniquely to the reference genome.

### Fluidigm genotyping

SNP-type oligonucleotide assays were designed by Fluidigm based on our genotyping information. Samples were genotyped using the EP1 platform on 96 x 96 plates following Fluidigm procedures (http://www.fluidigm.com).

### Genotype call

Signals were preprocessed to correct technical inaccuracies: 1) quantile normalization was applied between arrays and per probe normalizationby subtracting the median of the probes over arrays, 2) spatial slide defects, e.g., scratches, bubbles, etc., were removed by the method described in Chi et al. [36], and 3) calculating the average over allele probe replicates (forward and reverse) was performed by the ‘medianpolish’ method on a matrix of replicates x arrays to remove the array effect.

A training set was prepared by hybridizing gDNA from ‘9930’, ‘Gy14’, and their F_1_ progeny in six, five, and five replicates, respectively. On this array, the genotype calls are expected to be AA, AB, and BB for ‘9930’, F_1_, and ‘Gy14’ respectively. A K-mean clustering was applied to define three distinct clusters. Only probe sets that had three distinct clusters of the two parental lines and F_1_, or clusters that had one misclustered signal were used for genotype calling.

Genotype calling was performed by the Mahanalobis method [22]. Each SNP signal related to a sample in the test sets was assigned AA, AB, BB, or ‘no call’ if a call had a low confidence call. A confidence value was calculated for each genotype as follows: d0 is the Mahalanobis distance of a point to the center of its own type of call region, and d1 is the distance to the center of the closest region of the other type. The confidence of this call is d1/(d1 + d0).

### Linkage-map construction

Initially, we removed the noises that might affect the accuracy of the genetic map by: 1) leaving only one representative SNP for a set of SNPs whose genotype calls are exactly the same across all RILs, 2) filtering out SNPs with too many low-confidence calls, and 3) filtering out SNPs with observed heterozygous genotype frequencies that deviate from that expected in an F_7:8_ RIL population.

For groups of SNPs with redundant genotype calls, the SNP with the most distinct signal clusters was selected to represent the group. Distances between all pairs of the three cluster types were calculated by Bhattacharyya distance [38]. Each SNP had two distances among three clusters. The minimal distances were compared among the redundant SNPs and the SNP with the greatest distance was selected for the map. A confidence call value of 0.6 or less was defined as a low confidence call. The median of low confidence calls per SNP and median of absolute deviations (MAD) were calculated, and SNPs with a number of low confidence calls greater than 1 MAD unit above the median were filtered out. The last criterion for filtering was based on the expected genotype call distribution of SNPs along the RILs. In the F_7:8_ RIL population, the expected probabilities were 63/128, 2/128, and 63/128 for AA, AB, and BB, respectively. The frequency of genotype calls for each SNP was calculated and SNPs that deviated significantly from the expected distribution (χ^2^ test; *p*-value <0.05; Bonferroni correction for multiple testing) were filtered out.

SNP discovery was performed on version 1 of the ‘9930’ genome reference, which was available at the time. When the genome reference was updated to version 2, we removed SNP positions that were inconsistent between the two versions from the map. As a result, 209 additional SNPs were filtered out for a total of 11,156 markers.

Out of the initial 136 RIL samples that were hybridized on the array, two samples could not be normalized because of technical problems in the hybridization. In 10 samples, either the proportion of the heterozygous calls was higher than expected (0.2), or it was uninformative (homozygous calls were less than 0.15). Thus, data from 124 RILs were ultimately used in the linkage analysis. These data were submitted as input to ‘MSTMap’ program (http://alumni.cs.ucr.edu/~yonghui/mstmap.html) to construct a genetic map with the following parameters: **genetic distance** = halden, **cut_off_p_value** = 1e-10, **no_map_dist** = 15, **no_map_size** = 2, **missing_threshold** = 0.15, **estimation_before_clustering** = no, **detect_bad_data** = no, **objective_function** = ML. The outcome was a genetic map divided into bins where the start of each bin indicated the position of one or more SNP markers on the map. The order of the loci on the genetic map was used to refine unexpected genotype calls. As a result, an additional seven samples that had more than 10 ‘no call’ regions (described in the next section) were filtered out and a new linkage map of 117 RILs was constructed.

### Refining genotype calls

The improvement of genotype calls was performed in two stages—for regional and specific loci. Each locus call that was different from its flanking loci was modified by the following criteria. Where six or more flanking loci from both sides of the locus were called other than the locus call, but consecutively, this locus was modified to the call of the flanking loci. If the flanking loci did not contain uniformly consecutive calls, the locus call was set to ‘no call’. If a region was stretched out over 12 or more different consecutive calls, the whole region was set to ‘no call’.

### Estimation of local recombination frequency

Recombination rate is the slope of the genetic distance (cM) vs. the physical distance (Mb) for each linkage group against the chromosome under comparison (S1 and S2 Figs). Prior to slope computation, the genetic vs. physical position was smoothed by the Lowess method with span = 0.1. The local recombination rate was estimated by calculating the slope of five SNPs on the map in a running window.

### Segregation-distortion analysis

The 44,360 SNPs on the array were filtered by the criteria for redundant SNPs and for SNPs with too many low-confidence calls as described in the genotype call section. Genotype frequencies were calculated for each allele. The proportions of homozygous genotype calls, p(AA) and p(BB), were calculated per SNP. A spline non-linear regression of p(AA)–p(BB) was plotted against the genome position. A significant distortion from the allele segregation was calculated by chi-square test under the null hypothesis that the frequencies of p(AA) and p(BB) are equal and a Benjamini and Hochberg method [39] for multiple testing correction was applied.

## Results

### Genotyping-array design

Two properties should be taken into consideration when selecting a pool of SNPs for genotyping. Association studies ideally require that SNP markers cover the whole genome at high resolution, to decrease the chances of a recombination event between a SNP marker and the trait locus. The other property is the SNP marker's polymorphism in the segregating population under study. Commonly, enrichment with that type of SNP is achieved by selecting for SNPs that are polymorphic in a germplasm. Alternatively, by having a large pool of SNPs that cover the genome, one can expect to find a subset of SNPs that will be polymorphic and therefore informative in any association study.

Advanced algorithms for genotyping microarrays are based on machine learning techniques [40]. These algorithms use expected genotype calls as a training set to acquire the rules of genotyping, and then apply those rules to the test set under study [37,41]. The variance of genotype calls in microarrays is very high among SNPs [42]. Therefore, having the expected genotype calls for each SNP on the array is key for accurate genotyping. To satisfy this requirement, instead of collecting a set of polymorphic SNPs, our strategy was to define a pool of SNPs that has the expected genotype call in the training set, and use this set to design the array. This approach was implemented by discovering homozygous loci that are polymorphic between the two accessions, hereafter designated homozygous SNPs. The hybridization of the two accessions and their F_1_ progeny can be used as a training set in the genotyping as they are expected to show all three types, i.e., AA, AB, and BB for each SNP. We implemented this approach by mapping both the 24,761,994 long reads (454 GS-FLX Technology) of ‘Gy14’ and the 88,086,537 short reads (Illumina HiSeq-2000) of ‘9930’ to the reference genome of the latter [17]. SNPs were selected within 2-Kb windows, throughout the genome, based on the following priorities: “homozygous SNPs”, highest read depth, and highest quality. As a result, 44,360 SNPs were used to complete the 45K Agilent array, of which 36,779 were homozygous.

The coverage of most (90%) of the SNPs on the array was in the range of 8–39 reads per locus with a median allele A-to-allele B coverage ratio of 1:2. Table 1 summarizes the properties of the SNPs on the array across the seven chromosomes of the ‘9930’ and ‘Gy14’ [19] reference genomes. Fifteen percent of the SNPs that were used for the array design were located in coding sequences (CDSs). SNPs within CDSs are of interest because they might modify the amino acid composition of a protein. The number of SNPs within CDSs ranged from 562 and 514 on chromosome 5 to 1502 and 1425 on chromosome 3 for ‘9930’ and ‘Gy14’ genomes, respectively (Table 1). In general, 77% of all genes were represented by a SNP on the array with an average of 1.3 markers per CDS.

**Table 1:** Summary of marker properties on the array and in the 'good training set' subset.

		Chinese Long ‘9930’ (Version 2)					North American ‘Gy14’ (Version 1)			
Chr		Length (Mb)	# SNPs	# SNPs in CDS	# SNPs in mRNA	Median marker interval	Length (Mb)	# SNPs in CDS	# SNPs in mRNA	Median marker interval
**1**	In SNP Chip	29.1	7324	985	3293	1979	28.4	909	3080	1946.5
	In good training set		5806	780	2687	2170		729	2530	2140
**2**	In SNP Chip	23.2	4466	679	2166	2123	23.5	682	2220	2049
	In good training set		3584	518	1764	2252		525	1830	2191
**3**	In SNP Chip	39.8	10041	1502	4829	1962	40.3	1425	4571	1937
	In good training set		8254	1215	4054	2142		1147	3798	2116
**4**	In SNP Chip	23.4	5672	765	2638	2071	23.4	711	2478	2031
	In good training set		4056	528	1962	2326		506	1881	2268
**5**	In SNP Chip	28.0	4102	562	1658	2373	27.5	514	1578	2326
	In good training set		2680	349	1149	2588		341	1134	2522
**6**	In SNP Chip	29.1	6353	932	3055	2127.5	30.2	906	2981	2101
	In good training set		5131	729	2504	2306.5		712	2449	2295
**7**	In SNP Chip	19.2	4600	641	2061	2091	19.3	597	1949	2083
	In good training set		2789	357	1241	2491.5		352	1211	2451
**Sum**	In SNP Chip	191.9	42558	6066	19700	14726.5	192.6	5744	18857	14473.5
	In good training set		32300	4476	15361	16276		4312	14833	15983

### A subset of SNPs for genotype calling

**Fig 1:**
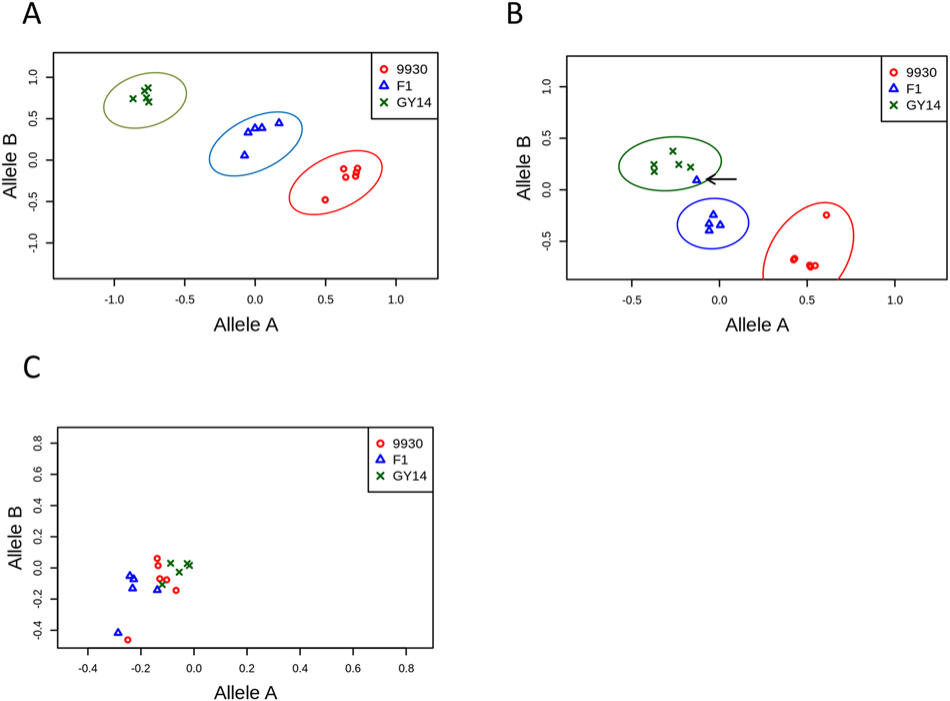
Example of three types of training sets. Hybridization signals of ‘GY14’ (green X), ‘9930’ (red circles), and their F_1_ (blue triangles) were plotted as scatter plots of allele X signals against allele Y signals. (A) Three distinct clusters were generated. This was considered a probe set for a SNP with a good training set. (B) Three distinct clusters were generated, one of which was clustered incorrectly. This was also considered a good training set. (C) No distinct cluster was generated. These SNP probe sets were not included in the genotype call analysis.

Agilent eArray technology enables generating a pool of probes and selecting a subset for future use. The criterion for probe-subset selection should rely on probes for which hybridization of the training set samples results in the expected genotype calls. Therefore, we performed hybridization with gDNA of the two parental lines (‘9930’ and ‘Gy14’) and their F_1_ progeny. The hybridizations of the three samples which resulted in the expected genotype calls generated a training set for each SNP. Out of the 44,360 SNPs on the array, a subset of 32,846 SNP-probe sets formed expected genotype calls or a good training set (GTS). A GTS was defined if three distinct clusters of allele-A signals against allele-B signals were identified by K-mean algorithm (Fig 1A). Moreover, we allowed a single signal in a SNP training set to be clustered incorrectly (Fig 1B). Only 9.8% of the SNPs that were defined as GTS had a single misclustered signal; the rest were perfectly clustered. We made the 32,846 GTS SNPs available as SNP arrays by splitting this set into two 15K Agilent arrays (Agilent design no. 066891 and 066895). A sample of 96 SNPs from the GTS was validated on the Fluidigm platform assayed on the three gDNA samples. A validated call was a match between the call on the Fluidigm and the equivalent call on the array, i.e., compatibility between technologies. Among the 96 SNPs, only 5 failed to have genotype calls for all replicated assays; the rest (91 Fluidigm calls) were 100% compatible with ‘9930’ and with F_1_, whereas 96% (87/91) were compatible with ‘Gy14’ array calls. In addition, a synthetic F_1_ was tested by mixing the gDNA of the two parental accessions that resulted in a 100% match to the genuine F_1_. This result suggested that a mixture of DNA from the two parental lines might be an alternative solution to extracting the gDNA of F_1_.

Narrowing the array to the GTS did not affect the proportion of SNPs in the CDS (Table 1), as it was proportional to the decrease in the number of markers in the subset (χ^2^ test, df = 1, *p*-values: 0.2–1; the range of *p*-values for all chromosomes for both genomes ‘9930’ and ‘Gy14’). Therefore, the GTS is a representative subset of the full set of SNPs on the array and using only the GTS subset should not significantly bias results.

### Genetic-map construction

**Fig 2:**
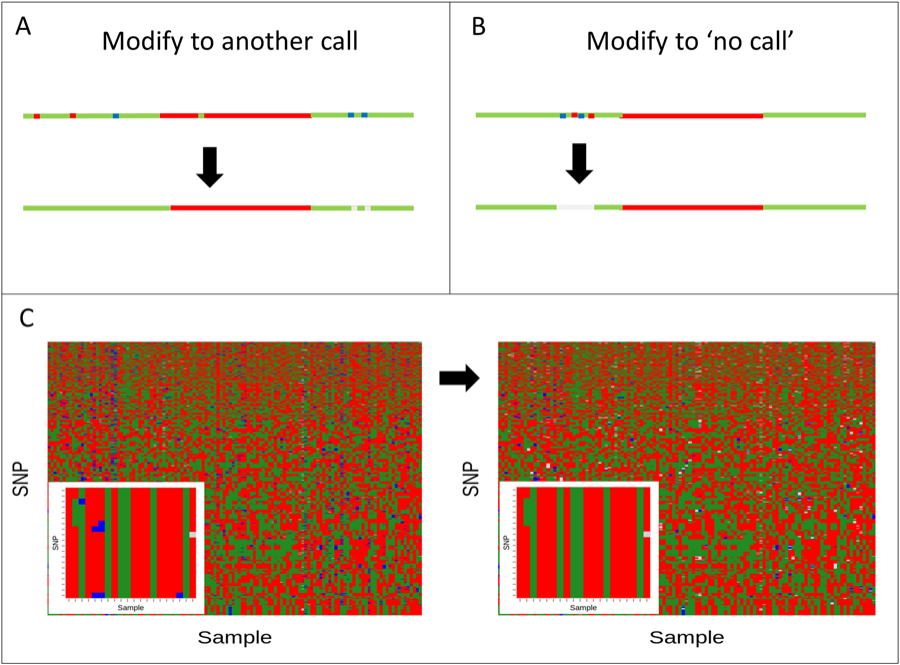
Illustration of genotype call refinement. Genotype call refinement was performed to improve the genotype call for stretches or blocks. Inconsistencies within a block were corrected to either the genotype block (AA—red; BB—green; AB—blue) or to no call (light gray). (A) An example of specific locus refinement by flanking loci according to the following criteria. First, six or more flanking loci from both sides of the locus were called other than the locus call, but consecutively. This locus was modified to the call of the flanking loci. Otherwise, if the flanking loci did not contain uniformly consecutive calls, the locus call was set to ‘no call’. (B) If a region was stretched out over 12 or more different consecutive calls, the whole region was set to ‘no call’. (C) A heatmap plot of the genotype calls, before and after refinement. To illuminate the improvement, a zoom into a subset of SNPs and RILs of 20 x 20 (left bottom corner) was plotted.

A genetic map based on 124 RILs was constructed using the GTS. When two or more SNPs had redundant genotype distribution along the RILs, only one was retained. SNPs with too many low-confidence genotype calls, SNPs with more heterozygous calls than expected, and uninformative SNPs were also removed. The genotype calls of the remaining SNPs were further improved by a stage of genotype call refinement based on the flanking vicinity in a haplotype block: after creating a genetic map, the genotype call was improved by modifying a call based on the flanking calls in a defined window (Fig 2). For example, a single AA call (homozygous to allele of ‘9930’) in a block of BB calls (homozygous to allele of 'Gy14') was changed to a BB call (Fig 2A), while a region of mixed AA, BB, and/or AB would get no calls (Fig 2A). This process (for details see Materials and Methods) created more homogeneous haplotype blocks (Fig 2C) and decreased the number of heterozygous calls from 2.5% to 1.5%, while increasing the number of ‘no call’ assignments from 0.24% to 0.5%. Consequently, the final linkage map was constructed based on 117 RILs that were retained after filtering out samples with more than 10 ‘no call’ regions. The genetic map included seven linkage groups of lengths 72.5, 87.1, 96.9, 104.7, 88.7, 87.0, and 61.8 for LG1–LG7, respectively (S1 Fig). The number of markers per linkage group was 2206, 1417, 2563, 1354, 860, 1709, and 1056 for LG1–7, respectively. Altogether, the map was constructed of 11,156 markers in 944 positions, and in many cases more than one marker was mapped to the same linkage-map position (bin). The median number of markers per bin was 13, 6, 12, 7.5, 4, 8, and 9 for LG1–7. The distribution was correlated with physical length. The exception was LG5, which contained the smallest number of markers despite a corresponding chromosome length greater than that of chromosome 2 or 7. The median interval between the map bins was 0.45 cM for all linkage groups (S1 Fig).

### Comparison of genetic and physical maps

**Fig 3:**
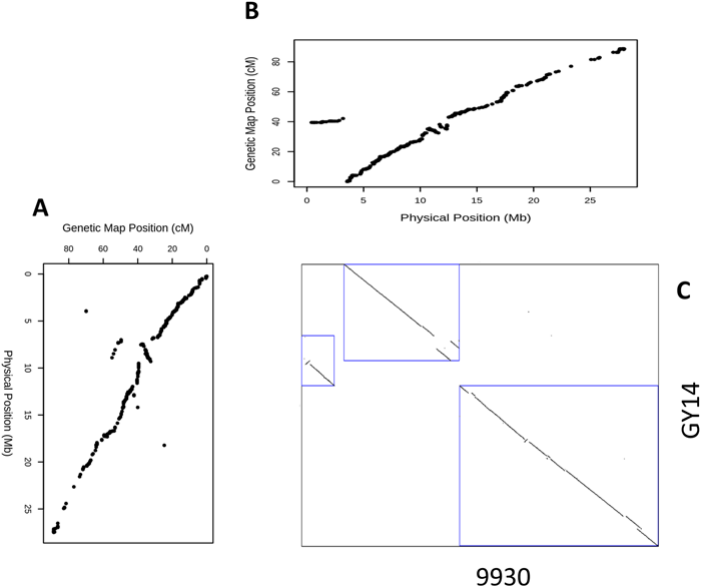
Genetic and physical map comparison for chromosome 5. Scatter plots of marker positions on the genetic map against the positions of the genome reference of both (A) ‘9930’ and (B) ‘Gy14’. These maps were drawn to their relative axis in a dot plot (C) of the two genomes for comparison.

To validate the order of the markers on the genetic map, we compared it to the genomes of both Chinese long ‘9930’ and the North American pickling type ‘Gy14’ (S2 and S3 Figs). Most of the markers (99.7%) were mapped to the chromosome that corresponded to their linkage group on the ‘9930’ reference genome. Similarly, 98.9% of the markers had a match between their linkage group and the corresponding 'Gy14' chromosome. The genetic map was highly correlated (R^2^ = 0.97 average for all chromosomes) with both genomes for all chromosomes, indicating its accuracy. However, there was a deviation from collinearity of a long segment of markers on LG5. This deviation appeared in the comparison of the genetic map to the ‘9930’ genome but not to the ‘Gy14’ genome (Fig 3). The putative translocation was supported by a comparison of the two genomes using SyMap [43] (S4 Fig). In addition to the six previously reported inversions [19] on chromosomes 4, 5, and 7, which were reconfirmed by SyMap comparison, new chromosomal rearrangements were observed. On chromosome 3, there were two duplications (0.3 Mb and 0.4 Mb long), as well as small duplications between chromosomes 3 and 2 (0.5 Mb long) and chromosomes 3 and 7 (0.5 Mb and 0.2 Mb long). Two inversions were observed on chromosome 4 (0.91 Mb and 1.55 Mb), three on chromosome 3 (0.63 Mb, 0.77 Mb, and 0.65 Mb) and one on chromosome 7 (0.66 Mb) (S4 Fig).

### Recombination rates

The average recombination rate per chromosome was 3.2 ± 0.8 cM/Mb. The highest estimated recombination rate was on chromosome 4 (4.6 cM/Mb) and the lowest on chromosome 3 (2.4 cM/Mb). Although these maximum and minimum values did not exceed the 95% confidence intervals (1.7–4.7 cM/Mb), the recombination rate on chromosome 4 was close to the upper bound of the confidence interval for the whole-genome recombination rate.

Recombination suppression has been previously reported on chromosomes 4, 5 and 7 in a segregating population of a cross between landrace cucumber accessions ‘PI 183967’ and ‘Gy14’ [23]. These suppression events were not observed in the ‘9930’ × ‘Gy14’ F_2_ population [19]. In the ‘9930’ × ‘Gy14’ RIL population, rather than recombination suppression, there seemed to be an increase in recombination rate on chromosome 4.

**Fig 4:**
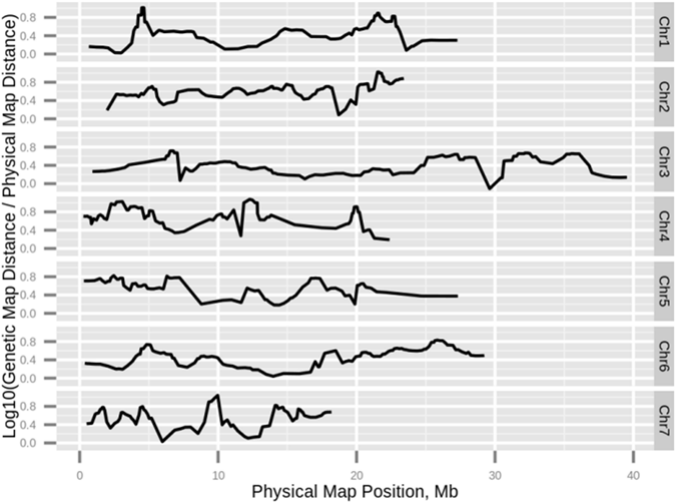
Regional recombination rate. For each chromosome, a sliding window was run over 5 SNP markers on the genetic map. Within that window, the slope of the genetic distance (cM) vs. physical genomic distance (Mb) was calculated and plotted on a log scale. Markers whose order on the genetic map was inconsistent with their order on the genome were removed. The recombination rate was calculated for the 'Gy14' genome.

An ultradense genetic map provides the opportunity to investigate the regional rate of recombination and explore whether the recombination rate is uniformly distributed. By calculating the standard deviation of the local recombination rate along the chromosomes, we can estimate whether the recombination rate is uniform along each chromosome, or variable, i.e., subjected to a local effect. The local recombination rate was calculated as the log genetic distance (cM) to thegenomic distance (Mb) ratio along five markers within a running window (Fig 4). The standard deviation of recombination rate on chromosome 4 was 0.26, which was 50% more variable than the standard deviation of recombination rate on the chromosome with the smallest regional effect (chromosome 2; 0.17). Thus, the higher rate on chromosome 4 could be attributed to local events around positions 12.5 Mb and 20 Mb. Chromosomal rearrangements are known to affect recombination rate [44]. However no positional associations between chromosomal rearrangements (S4 Fig) and recombination rates were observed, suggesting that additional factors might be influencing the recombination rate.

### Segregation distortion

**Fig 5:**
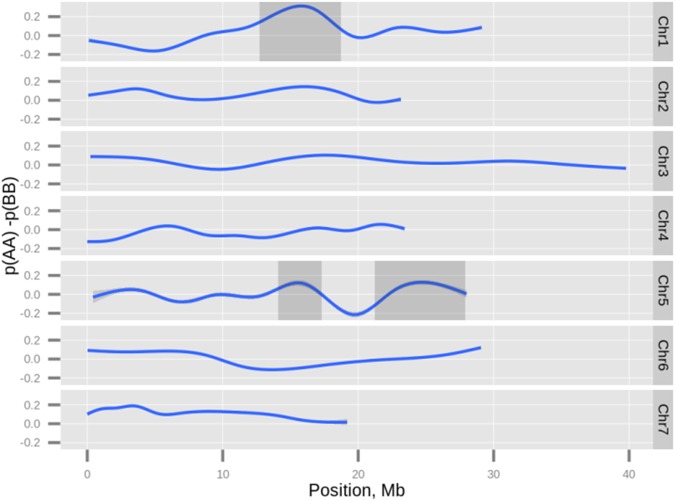
Differential genotype proportion. The proportions of homozygous genotype calls p(AA) and p(BB) were calculated. A spline non-linear regression of p(AA)–p(BB) was plotted against the genome position. The genomic areas of segregation distortion (gray rectangles) were estimated by chi-square test under the null hypothesis that the frequencies of p(AA) and p(BB) were equal. The null hypothesis was rejected at a level of 5% adjusted by Benjamini-Hochberg method for false discovery rate.

Segregation distortion has been suggested to occur more frequently in recombinant inbred populations than in F_2_ population [45]. Genetic factors suggested to affect segregation distortion are preferential fertilization, pollen lethality, and chromosomal translocation [46]. Local segregation-distortion regions (SDRs) may be observed by calculating the deviation of allele proportions from that expected on F_7:8_ of the RIL population. Three SDRs were observed, one on chromosome 1 (~70%) and two on chromosome 5 (Fig 5). The putative translocation of a 2.59 Mbp region on chromosome 5 (starting at 9.27 Mb on the ‘Gy14’ genome) occurred before the first SDR on chromosome 5. Unless there are *cis* effects, the translocation itself cannot explain the segregation distortion. The direction of the distorted segregation in all three cases favored the ‘9930’ allele (A-allele) (Fig 5).

### SNPs in other cucumber lines

**Fig 6:**
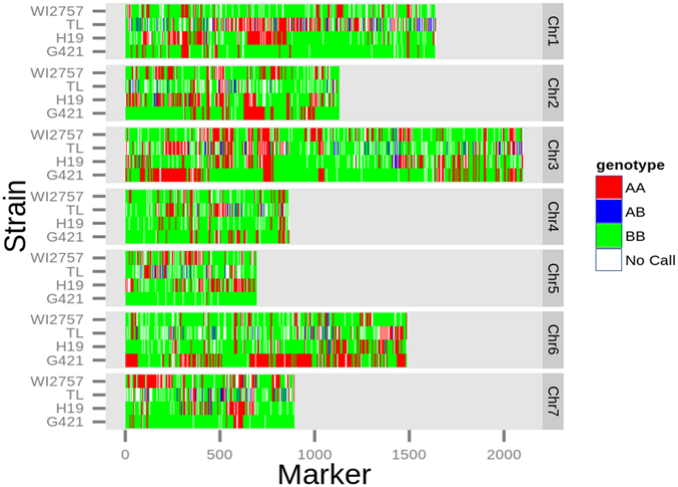
Application of cucumber genotyping array to four cucumber accessions. Genomic DNA of four cucumber accessions—H19, TL, G421, and WI 2757—was hybridized on the cucumber array. The genotype calls of the array were plotted as heatmap per chromosome where A is the ‘9930’ allele and B is the ‘Gy14’ allele. The mixed color blocks illustrate the polymorphic property of the SNPs on the array.

To illustrate the applicability of this cucumber SNP array, we used it to genotype four inbred lines of cultivated cucumber: 'H19', 'TL', 'G421', and 'WI 2757'. Out of the 32,846 GTS SNPs, 93% were successfully genotyped (repeated in duplicate) in two out of the four accessions, and 77% in three accessions. Forty-seven percent of the SNPs on the linkage map were not polymorphic (Fig 6; green and red blocks in four variants). However, out of 11,165 markers on the map, 10,709 (96%) fell in 774 out 994 bins (82%), of which at least one of the markers in those bins was polymorphic. Thus, 82% of the genetic map was covered by polymorphic bins. For example, on LG1, the seventh bin at position 3.2 cM was comprised of 36 SNPs, 5 of which were non-polymorphic while the other 31 were polymorphic among those accessions. When we examined the 5,319 non-polymorphic SNPs, 4,863 of them were in a bin that included polymorphic SNPs as well. In other words, there is a 91% chance of replacing a non-polymorphic SNP by another linked polymorphic SNP in one of the four non-parental accessions.

## Discussion

Since the first release of the whole-genome sequence of cucumber, many studies have been conducted to create and improve the resolution its genetic maps. These maps were primarily based on SSRs or on combinations of SSRs and SNPs [19,23,47] and did not lead to the development of a high-throughput tool for massive parallel genotyping. Such a tool would be useful to re-genotype many other cucumber populations. In this study, we provide the first report, to the best of our knowledge, of the development of a custom dual-color genotyping microarray based on Agilent technology. The dual-channel platform saves on slides by genotyping two individuals on one slide. We illustrate its application by creating a cucumber genetic map and comparing it with two cucumber genomes, as well as by genotyping other cucumber accessions.

One of the features of SNP arrays is separation of the SNP discovery and genotyping steps. In the first step, a pool of SNPs is optimized for polymorphic properties and successful genotyping calls; in the second step, the array is used recurrently by the community studying a particular organism. As a result, the reference SNPs enable sharing the results among different studies [9,10,15]. In contrast, GBS bundles the two steps, and a new set of SNPs are discovered and genotyped for each study. Sharing GBS results among studies is inferior to use of the array, and the intersection of SNPs among these studies might be small. The intersection will depend on the amount of successfully genotyped SNPs, which in turn depends on restriction-enzyme optimization and high coverage [62]. In contrast, GBS is much more cost-effective than SNP arrays. Even though the effective SNP number may be smaller from GBS than from the SNP array, the number of markers may still be powerful enough for successful mapping of quantitative trait loci (QTL) [62].

### Array design and accuracy

In previous studies, selection of SNPs was based on their polymorphic properties in the germplasm of the organism [6,9,13,15], as well as on enriching for SNPs in genic regions [9]. Most of those arrays were designed for the Illumina platform, which discriminates alleles by ligation and primer extension. In microarray platforms like Affymetrix and Agilent, the allelic discrimination is performed by hybridization. These platforms require a training set for accurate genotyping. Therefore, we focused our array design on SNPs that will have a GTS and also cover the whole cucumber genome. One of the features of a training set is that it requires gDNA hybridization of the two parents and their F_1_ hybrid; however F_1_ hybrids may not always be available. We show here that the F_1_ gDNA can be replaced with synthetic F_1_ that is a mixture of the two accessions' DNA. A possible pitfall to this strategy may lie in the fact that we did not select primarily for SNP markers that are polymorphic in the available cucumber germplasm collection [29,48]. However, given the large number of SNP markers (32,864), one might expect to find markers that will be polymorphic in any segregating population. This fact was illustrated by successfully genotyping several other cucumber inbred lines (Fig 6) using our GTS data (deposited in GEO; accession no. GSE57294). In these accessions, only 53% of the total SNPs on the map were polymorphic; however, four accessions constitute only a small sample of the cucumber germplasm [29,48], and therefore one would expect that the number of polymorphic SNPs will increase as the number of accessions included in the study increases. In comparison, other organism studies have reported a similar percentage of polymorphic SNPs in their arrays. The maize SNP array reported more than 40% polymorphic SNPs in segregating populations other than the one for which the array was designed [6], and in the RiceSNP50 array, 60% of the SNPs were informative in other variants [9]. Another work that designed a peach SNP-array reported 57% polymorphic SNPs in the germplasm that was used for array validation [13]. Moreover, we showed that because the genetic map is very dense and there is more than one SNP per bin, 91% of the non-polymorphic SNPs can be replaced by polymorphic ones sharing the same bin. As a result, 82% of the bins were polymorphic in one of the non-parental accessions. That result supports the strategy of constructing a large pool of SNPs that cover the genome (on average every 2 Kb) as an alternative to focusing on polymorphic SNPs in a germplasm. The rationale for favoring the former strategy is that the accuracy of genotyping can be controlled, whereas SNP polymorphism cannot.

### Genotyping-array applications

Application of the array was illustrated by developing an ultrahigh-density linkage map and comparing it with two cucumber genome references. Previous studies reported genetic maps of 572.9 and 707.8 cM in length with 678 and 735 bins and average intervals of 0.84 cM and 0.96 cM, respectively. The former map was derived from a segregating population of ‘Gy14’ × ‘PI183967’ [23] and the latter from ‘Gy14’ × ‘9930’ [19]. The total length of the genetic map constructed in this study falls in the middle and is based on a denser map than the previous ones (grand mean of 0.64 cM). Moreover, in our map, we eliminated some of the unexpected heterozygous loci by a process of refinement. This might improve the map’s accuracy. However, it may also eliminate possible gene-conversion events [49,50].

In general, linkage group lengths were correlated with their physical lengths. LG4 was an interesting exception. In three different maps, based on ‘Gy14’ × ‘PI183967’ RILs, ‘Gy14’ × ‘9930’ F_2_, and in this study ‘Gy14’ × ‘9930’ RILs, the length of LG4 was estimated differently. In the first map, it was estimated to be shorter than the corresponding physical length. This led to the conclusion that a recombination-suppression event had occurred. This recombination suppression was not observed in the second map, possibly indicating that the recombination event was not in the lineage of the common accession between the two maps, i.e., ‘Gy14’. In the last estimation of LG4, in the ‘Gy14’ × ‘9930’ RIL map, LG4's length was higher than its corresponding physical length. The difference in recombination rate between the two ‘Gy14’ × ‘9930’ maps might be due to differences in the markers and densities of the maps. The estimation of global rate in all three cases was done globally, i.e., the chromosomal recombination rate divided by the physical length of the chromosome. We showed that the recombination rate is not uniform along the chromosome, especially on chromosomes 1, 7, and 4. Therefore, estimation of the global recombination rate based on a low-density linkage map might be misleading. The areas of low recombination might be associated with selection and may cause a reduction in genomic variation [51]. In contrast, the areas of high recombination suggest the existence of hot-spots for recombination [52]. Thus recombination-rate estimation based on a low number of markers might skip sensing the local recombination hot-spot and suppression, resulting in misleading conclusions.

The genetic map generated by the cucumber genotyping array was highly correlated to both reference genomes. Although the positions of the SNPs were derived from the ‘9930’ reference genome, the genetic map was more correlated to the ‘Gy14’ genome. This might indicate a better draft for the ‘Gy14’ genome than for the ‘9930’ reference genome. An exception to the high correlation between the genetic map and the physical map was LG5. A segment of 2.6 Mb from the beginning of chromosome 5 of ‘9930’ fell in the middle of the genetic map. Note that some of the genomic rearrangements, such as six inversions, have been reported and validated by in situ techniques [19]. These results should be regarded with caution pending further validation, as the translocations and duplications were detected by bioinformatics tools and might result from incorrect assemblies.

Distortion of allele segregation has been previously reported on cucumber chromosomes 1, 4 and 6 in favor of the ‘PI183967’ parent [23]. Preference for one parental allele in SDRs was observed, in this work, for the male parent, i.e., the ‘9930’ allele. Preferential segregation for one parent, either male or female, has been reported for maize [53] on a chromosomal scale, and in rice [45] on a smaller scale. Although in this study, the observed distortion favored the male parent, this might not always be the case [54].

Complex traits such as leaf size and resistance to powdery mildew disease have been mapped on ‘H19’ × ‘G421’ and ‘WI 2575’ × ‘TL’ segregating populations respectively [27,55]. High-density maps are key for accurate QTL mapping. Our mapping efforts of QTL for fruit length (unpublished data) suggest that the cucumber genotyping array will be useful in mapping other cucumber traits, such as fruit weight, fruit length, and fruit flesh thickness [28,56].

## Conclusions

We developed a genotyping array based on a large set of SNPs discovered as a variation between two parental lines of cucumber whose genomes have already been sequenced. Instead of enriching the genotyping array with polymorphic SNPs, our strategy was to enrich the array with SNPs that can be accurately genotyped, and increase the number of successful calls. The SNPs on the array were selected as polymorphic but homozygous between two parents and hybridizations of those parents’ gDNA and their F_1_ progeny were used to generate expected genotype calls as a training set. Such a tool is powerful for future cucumber genetic studies, especially where a large number of markers is beneficial. We suggest that the large number of markers is key for marker polymorphism transferability to other lines. An ultra-dense linkage map constructed by this genotyping SNP array on a RIL population is expected to be invaluable for improving assembly and for mapping important QTL. Moreover, new knowledge of recombination rate and segregation distortion may contribute to cucumber genetic studies. Markers that deviate from Mendelian segregation are either removed while performing linkage-map construction [57] or a correction in the model is required [58]. In this linkage map, we removed those SNPs; however, if better resolution is needed in the future for SDRs, the other option can be used. Recombination rate has been shown to be correlated with sequence parameters [59] and with the rate of genetic variations [60,61]. Thus, the local rates of recombination reveal regions on the linkage groups where the rate of evolution is of interest.

## Supporting Information

S1 Fig Cucumber genetic map based on ‘Gy14’ x ‘9930’ RIL population. Genetic map was constructed with 117 ‘Gy14’ x ‘9930’ F8 RILs and 11,156 SNP markers. For simplicity, only 994 markers were drawn on this map using MapChart, i.e., a representative marker for each bin.
(TIF)

S2 Fig ‘Gy14’ x ‘9930’ genetic map vs. ‘Gy14’ draft genome (V1.0). Scatter plots of marker positions on the genetic map against their physical locations in the ‘Gy14’ genome (version 1.0). The order of the markers on the genetic map is highly collinear with their order in the ‘Gy14’ genome. The R2 values for chromosomes 1 through 7 are 0.99, 0.96, 0.99, 0.98, 0.96, 0.96, and 0.97, respectively.
(TIF)

S3 Fig ‘Gy14’ x ‘9930’ genetic map vs. ‘9930’ draft genome (V2.0). Scatter plots of marker positions on the genetic map against their positions in the ‘9930’ genome (version 2.0). The order of the markers on the genetic map is collinear with their order in the genome. The R2 values for chromosomes 1 through 7 are 0.99, 0.99, 0.99, 0.99, 0.82, 0.96, and 0.98, respectively.
(TIF)

S4 Fig Dot plot of the ‘Gy14’ (V1.0) vs. ‘9930’ (V2.0) genomes with SyMap Version 4. (TIF)

## References

[1] Xie W, Feng Q, Yu H, Huang X, Zhao Q, Xing Y, et al Parent-independent genotyping for constructing an ultrahigh-density linkage map based on population sequencing. Proc Natl Acad Sci. 2010;107:10578–10583. 10.1073/pnas.1005931107 20498060 PMC2890813

[2] Sim S-C, Durstewitz G, Plieske J, Wieseke R, Ganal MW, Van Deynze A, et al Development of a Large SNP Genotyping Array and Generation of High-Density Genetic Maps in Tomato. PLoS ONE. 2012;7:e40563 10.1371/journal.pone.0040563 22802968 PMC3393668

[3] Xu Y, Lu Y, Xie C, Gao S, Wan J, Prasanna BM. Whole-genome strategies for marker-assisted plant breeding. Mol Breed. 2012;29:833–854. 10.1007/s11032-012-9699-6

[4] Khan MA, Korban SS. Association mapping in forest trees and fruit crops. J Exp Bot. 2012;63:4045–4060. 10.1093/jxb/ers105 22511806

[5] Collard BCY, Mackill DJ. Marker-assisted selection: an approach for precision plant breeding in the twenty-first century. Philos Trans R Soc B Biol Sci. 2008;363:557–572. 10.1098/rstb.2007.2170 17715053 PMC2610170

[6] Yan J, Yang X, Shah T, Sánchez-Villeda H, Li J, Warburton M, et al High-throughput SNP genotyping with the GoldenGate assay in maize. Mol Breed. 2010;25:441–451. 10.1007/s11032-009-9343-2

[7] Alkan C, Coe BP, Eichler EE. Genome structural variation discovery and genotyping. Nat Rev Genet. 2011;12:363–376. 10.1038/nrg2958 21358748 PMC4108431

[8] Gupta PK, Rustgi S, Mir RR. Array-based high-throughput DNA markers for crop improvement. Heredity. 2008;101:5–18. 10.1038/hdy.2008.35 18461083

[9] Chen H, Xie W, He H, Yu H, Chen W, Li J, et al A High-Density SNP Genotyping Array for Rice Biology and Molecular Breeding. Mol Plant. 2013;sst135 10.1093/mp/sst135 24121292

[10] Pavy N, Gagnon F, Rigault P, Blais S, Deschênes A, Boyle B, et al Development of high-density SNP genotyping arrays for white spruce (Picea glauca) and transferability to subtropical and nordic congeners. Mol Ecol Resour. 2013;13:324–336. 10.1111/1755-0998.12062 23351128

[11] Miller AJ, Matasci N, Schwaninger H, Aradhya MK, Prins B, Zhong G-Y, et al Vitis Phylogenomics: Hybridization Intensities from a SNP Array Outperform Genotype Calls. PLoS ONE. 2013;8:e78680 10.1371/journal.pone.0078680 24236035 PMC3827278

[12] Antanaviciute L, Fernández-Fernández F, Jansen J, Banchi E, Evans KM, Viola R, et al Development of a dense SNP-based linkage map of an apple rootstock progeny using the Malus Infinium whole genome genotyping array. BMC Genomics. 2012;13:203 10.1186/1471-2164-13-203 22631220 PMC3410780

[13] Verde I, Bassil N, Scalabrin S, Gilmore B, Lawley CT, Gasic K, et al Development and Evaluation of a 9K SNP Array for Peach by Internationally Coordinated SNP Detection and Validation in Breeding Germplasm. PLoS ONE. 2012;7:e35668 10.1371/journal.pone.0035668 22536421 PMC3334984

[14] Sharpe AG, Ramsay L, Sanderson L-A, Fedoruk MJ, Clarke WE, Li R, et al Ancient orphan crop joins modern era: gene-based SNP discovery and mapping in lentil. BMC Genomics. 2013;14:192 10.1186/1471-2164-14-192 23506258 PMC3635939

[15] McCouch SR, Zhao K, Wright M, Tung C-W, Ebana K, Thomson M, et al Development of genome-wide SNP assays for rice. Breed Sci. 2010;60:524–535.

[16] Zhao K, Tung C-W, Eizenga GC, Wright MH, Ali ML, Price AH, et al Genome-wide association mapping reveals a rich genetic architecture of complex traits in Oryza sativa. Nat Commun. 2011;2:467 10.1038/ncomms1467 21915109 PMC3195253

[17] Huang S, Li R, Zhang Z, Li L, Gu X, Fan W, et al The genome of the cucumber, Cucumis sativus L. Nat Genet. 2009;41:1275–1281. 10.1038/ng.475 19881527

[18] Arumuganathan K, Earle ED. Nuclear DNA content of some important plant species. Plant Mol Biol Report. 1991;9:208–218. 10.1007/BF02672069

[19] Yang L, Koo D-H, Li Y, Zhang X, Luan F, Havey MJ, et al Chromosome rearrangements during domestication of cucumber as revealed by high-density genetic mapping and draft genome assembly. Plant J. 2012;71:895–906. 10.1111/j.1365-313X.2012.05017.x 22487099

[20] Wóycicki R, Witkowicz J, Gawroński P, Dąbrowska J, Lomsadze A, Pawełkowicz M, et al The Genome Sequence of the North-European Cucumber (Cucumis sativus L.) Unravels Evolutionary Adaptation Mechanisms in Plants. PLoS ONE. 2011;6:e22728 10.1371/journal.pone.0022728 21829493 PMC3145757

[21] Li D, Cuevas HE, Yang L, Li Y, Garcia-Mas J, Zalapa J, et al Syntenic relationships between cucumber (Cucumis sativus L.) and melon (C. melo L.) chromosomes as revealed by comparative genetic mapping. BMC Genomics. 2011;12:396 10.1186/1471-2164-12-396 21816110 PMC3199783

[22] Yang L, Koo D-H, Li D, Zhang T, Jiang J, Luan F, et al Next-generation sequencing, FISH mapping and synteny-based modeling reveal mechanisms of decreasing dysploidy in Cucumis. Plant J. 2014;77:16–30. 10.1111/tpj.12355 24127692

[23] Ren Y, Zhang Z, Liu J, Staub JE, Han Y, Cheng Z, et al An Integrated Genetic and Cytogenetic Map of the Cucumber Genome. PLoS ONE. 2009;4:e5795 10.1371/journal.pone.0005795 19495411 PMC2685989

[24] Cavagnaro PF, Senalik DA, Yang L, Simon PW, Harkins TT, Kodira CD, et al Genome-wide characterization of simple sequence repeats in cucumber (Cucumis sativus L.). BMC Genomics. 2010;11:569 10.1186/1471-2164-11-569 20950470 PMC3091718

[25] Zhang W-W, Pan J-S, He H-L, Zhang C, Li Z, Zhao J-L, et al Construction of a high density integrated genetic map for cucumber (Cucumis sativus L.). Theor Appl Genet. 2012;124:249–259. 10.1007/s00122-011-1701-x 21971891

[26] Yang L, Li D, Li Y, Gu X, Huang S, Garcia-Mas J, et al A 1,681-locus consensus genetic map of cultivated cucumber including 67 NB-LRR resistance gene homolog and ten gene loci. BMC Plant Biol. 2013;13:53 10.1186/1471-2229-13-53 23531125 PMC3626583

[27] Fazio G, Staub JE, Stevens MR. Genetic mapping and QTL analysis of horticultural traits in cucumber (Cucumis sativus L.) using recombinant inbred lines. Theor Appl Genet. 2003;107:864–874. 10.1007/s00122-003-1277-1 12827247

[28] Yuan XJ, Li XZ, Pan JS, Wang G, Jiang S, Li XH, et al Genetic linkage map construction and location of QTLs for fruit-related traits in cucumber. Plant Breed. 2008;127:180–188. 10.1111/j.1439-0523.2007.01426.x

[29] Qi J, Liu X, Shen D, Miao H, Xie B, Li X, et al A genomic variation map provides insights into the genetic basis of cucumber domestication and diversity. Nat Genet. 2013;45:1510–1515. 10.1038/ng.2801 24141363

[30] Knerr LD, Staub JE, Holder DJ, May BP. Genetic diversity in Cucumis sativus L. assessed by variation at 18 allozyme coding loci. Theor Appl Genet. 1989;78:119–128. 10.1007/BF00299764 24227040

[31] Dijkhuizen A, Kennard WC, Havey MJ, Staub JE. RFLP variation and genetic relationships in cultivated cucumber. Euphytica. 1996;90:79–87. 10.1007/BF00025163

[32] Li Y, Yang L, Pathak M, Li D, He X, Weng Y. Fine genetic mapping of cp: a recessive gene for compact (dwarf) plant architecture in cucumber, Cucumis sativus L. Theor Appl Genet. 2011;123:973–983. 10.1007/s00122-011-1640-6 21735235

[33] Murray MG, Thompson WF. Rapid isolation of high molecular weight plant DNA. Nucleic Acids Res. 1980;8:4321–4326. 10.1093/nar/8.19.4321 7433111 PMC324241

[34] Gresham D, Curry B, Ward A, Gordon DB, Brizuela L, Kruglyak L, et al Optimized detection of sequence variation in heterozygous genomes using DNA microarrays with isothermal-melting probes. Proc Natl Acad Sci. 2010;107:1482–1487. 10.1073/pnas.0913883107 20080586 PMC2824413

[35] SantaLucia J. A unified view of polymer, dumbbell, and oligonucleotide DNA nearest-neighbor thermodynamics. Proc Natl Acad Sci. 1998;95:1460–1465. Available: http://www.pnas.org/content/95/4/1460 9465037 10.1073/pnas.95.4.1460 PMC19045

[36] Chai HS, Therneau TM, Bailey KR, Kocher J-PA. Spatial normalization improves the quality of genotype calling for Affymetrix SNP 6.0 arrays. BMC Bioinformatics. 2010;11:356 10.1186/1471-2105-11-356 20587065 PMC2910027

[37] Rabbee N, Speed TP. A genotype calling algorithm for affymetrix SNP arrays. Bioinformatics. 2005;22:7–12. 10.1093/bioinformatics/bti741 16267090

[38] Kailath T. The Divergence and Bhattacharyya Distance Measures in Signal Selection. IEEE Trans Commun Technol. 1967;15:52–60. 10.1109/TCOM.1967.1089532

[39] Benjamini Y, Hochberg Y. Controlling the false discovery rate: a practical and powerful approach to multiple testing. J R Stat Soc Ser B Methodol. 1995;289–300.

[40] Lin S, Carvalho B, Cutler D, Arking DE, Chakravarti A, Irizarry RA. Validation and extension of an empirical Bayes method for SNP calling on Affymetrix microarrays. Genome Biol. 2008;9:R63 Available: http://www.biomedcentral.com/content/pdf/gb-2008-9-4-r63.pdf 10.1186/gb-2008-9-4-r63 18387188 PMC2643934

[41] Lin et al.- 2008—Validation and extension of an empirical Bayes met.pdf. 10.1186/gb-2008-9-4-r63 PMC2643934 18387188

[42] Carvalho BS, Louis TA, Irizarry RA. Quantifying uncertainty in genotype calls. Bioinformatics. 2010;26:242–249. 10.1093/bioinformatics/btp624 19906825 PMC2804295

[43] Soderlund C, Bomhoff M, Nelson WM. SyMAP v3.4: a turnkey synteny system with application to plant genomes. Nucleic Acids Res. 2011;39:e68 10.1093/nar/gkr123 21398631 PMC3105427

[44] Zhang L, Wang S, Li H, Deng Q, Zheng A, Li S, et al Effects of missing marker and segregation distortion on QTL mapping in F2 populations. Theor Appl Genet. 2010;121:1071–1082. 10.1007/s00122-010-1372-z 20535442

[45] Xu Y, Zhu L, Xiao J, Huang N, McCouch SR. Chromosomal regions associated with segregation distortion of molecular markers in F2, backcross, doubled haploid, and recombinant inbred populations in rice (Oryza sativa L.). Mol Gen Genet MGG. 1997;253:535–545. 10.1007/s004380050355 9065686

[46] Liu X, Guo L, You J, Liu X, He Y, Yuan J, et al Progress of Segregation Distortion in Genetic Mapping of Plants. Res J Agron. 2010;4:78–83. 10.3923/rjagr.2010.78.83

[47] Han Y, Zhang Z, Huang S, Jin W. An integrated molecular cytogenetic map of Cucumis sativus L. chromosome 2. BMC Genet. 2011;12:18 10.1186/1471-2156-12-18 21272311 PMC3039625

[48] Lv J, Qi J, Shi Q, Shen D, Zhang S, Shao G, et al Genetic Diversity and Population Structure of Cucumber (Cucumis sativus L.). PLoS ONE. 2012;7:e46919 10.1371/journal.pone.0046919 23071663 PMC3470563

[49] Galtier N, Piganeau G, Mouchiroud D, Duret L. GC-Content Evolution in Mammalian Genomes: The Biased Gene Conversion Hypothesis. Genetics. 2001;159:907–911. Available: http://www.genetics.org/content/159/2/907 11693127 10.1093/genetics/159.2.907 PMC1461818

[50] Mondragon-Palomino M, Gaut BS. Gene Conversion and the Evolution of Three Leucine-Rich Repeat Gene Families in Arabidopsis thaliana. Mol Biol Evol. 2005;22:2444–2456. 10.1093/molbev/msi241 16120808

[51] Nachman MW. Variation in recombination rate across the genome: evidence and implications. Curr Opin Genet Dev. 2002;12:657–663. 10.1016/S0959-437X(02)00358-1 12433578

[52] Drouaud J, Camilleri C, Bourguignon P-Y, Canaguier A, Bérard A, Vezon D, et al Variation in crossing-over rates across chromosome 4 of Arabidopsis thaliana reveals the presence of meiotic recombination “hot spots.” Genome Res. 2006;16:106–114. 10.1101/gr.4319006 16344568 PMC1356134

[53] Lu H, Romero-Severson J, Bernardo R. Chromosomal regions associated with segregation distortion in maize. Theor Appl Genet. 2002;105:622–628. 10.1007/s00122-002-0970-9 12582513

[54] Kumar S, Gill BS, Faris JD. Identification and characterization of segregation distortion loci along chromosome 5B in tetraploid wheat. Mol Genet Genomics MGG. 2007;278:187–196. 10.1007/s00438-007-0248-7 17520291

[55] He X, Li Y, Pandey S, Yandell BS, Pathak M, Weng Y. QTL mapping of powdery mildew resistance in WI 2757 cucumber (Cucumis sativus L.). Theor Appl Genet. 2013;126:2149–2161. 10.1007/s00122-013-2125-6 23689747

[56] Yuan XJ, Pan JS, Cai R, Guan Y, Liu LZ, Zhang WW, et al Genetic mapping and QTL analysis of fruit and flower related traits in cucumber (Cucumis sativus L.) using recombinant inbred lines. Euphytica. 2008;164:473–491. 10.1007/s10681-008-9722-5

[57] Zhu C, Wang C, Zhang Y-M. Modeling segregation distortion for viability selection I. Reconstruction of linkage maps with distorted markers. Theor Appl Genet. 2007;114:295–305. 10.1007/s00122-006-0432-x 17119913

[58] Manly KF, Robert H. Cudmore J, Meer JM. Map Manager QTX, cross-platform software for genetic mapping. Mamm Genome. 2001;12:930–932. 10.1007/s00335-001-1016-3 11707780

[59] Groenen MAM, Wahlberg P, Foglio M, Cheng HH, Megens H-J, Crooijmans RPMA, et al A high-density SNP-based linkage map of the chicken genome reveals sequence features correlated with recombination rate. Genome Res. 2009;19:510–519. 10.1101/gr.086538.108 19088305 PMC2661806

[60] Farkhari M, Lu Y, Shah T, Zhang S, Naghavi MR, Rong T, et al Recombination frequency variation in maize as revealed by genomewide single-nucleotide polymorphisms. Plant Breed. 2011;130:533–539. 10.1111/j.1439-0523.2011.01866.x

[61] Gaut BS, Wright SI, Rizzon C, Dvorak J, Anderson LK. Recombination: an underappreciated factor in the evolution of plant genomes. Nat Rev Genet. 2007;8:77–84. 10.1038/nrg1970 17173059

[62] Beissinger TM, Hirsch CN, Sekhon RS, Foerster JM, Johnson JM, Muttoni G, et al Marker Density and Read Depth for Genotyping Populations Using Genotyping-by-Sequencing. Genetics. 2013;193:1073–1081. 10.1534/genetics.112.147710 23410831 PMC3606087

