# Supplementary figures and images for: Ultrahigh-Density Linkage Map for Cultivated Cucumber (Cucumis sativus L.) Using a Single-Nucleotide Polymorphism Genotyping Array
Source: PLoS One. 2015 Apr 13;10(4):e0124101. doi: 10.1371/journal.pone.0124101 (PMC4395401; doi:10.1371/journal.pone.0124101)

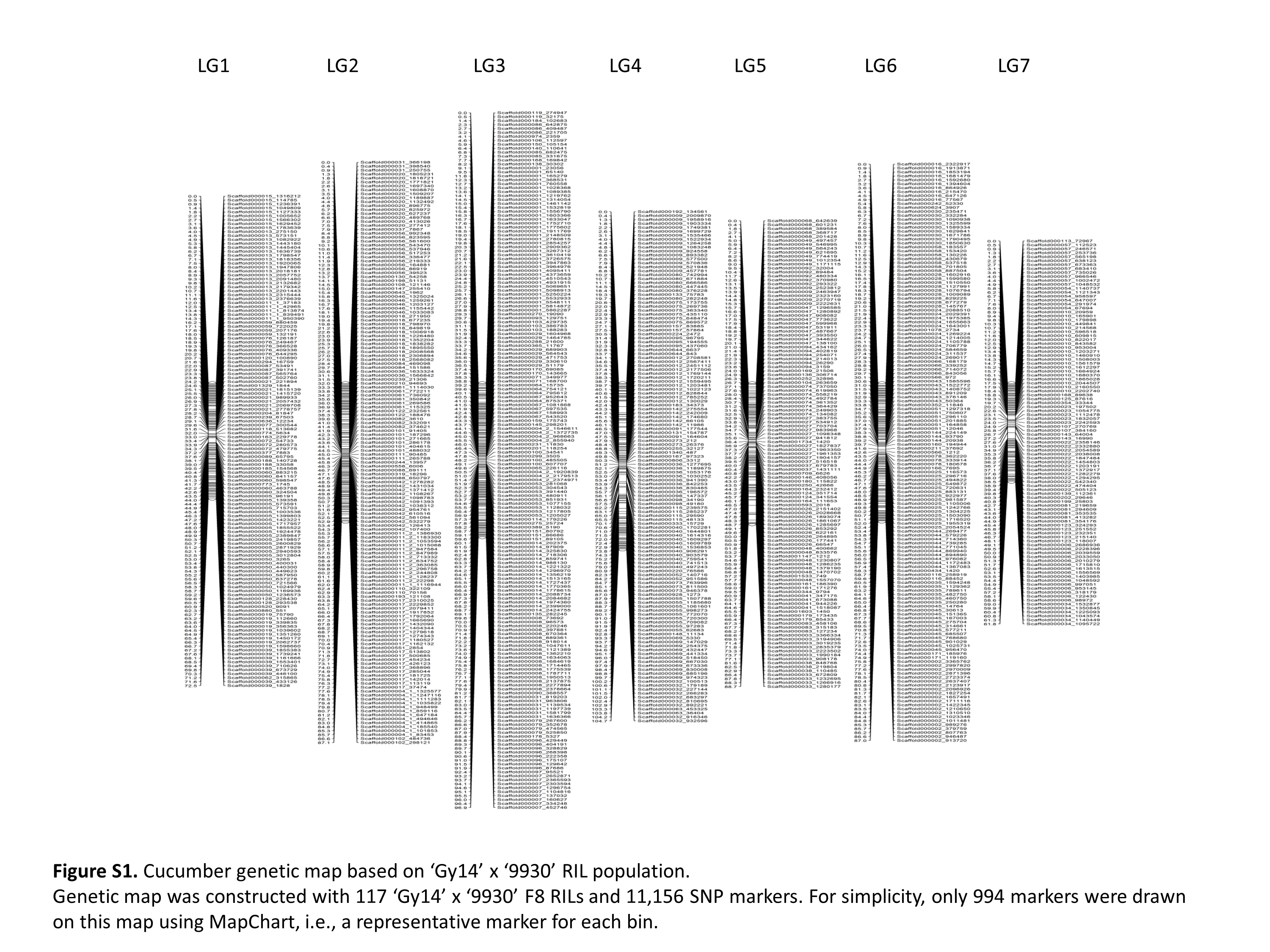

Supplement: S1 Fig — Genetic map was constructed with 117 ‘Gy14’ x ‘9930’ F8 RILs and 11,156 SNP markers. For simplicity, only 994 markers were drawn on this map using MapChart, i.e., a representative marker for each bin. (TIF) [file pone.0124101.s001.tif]

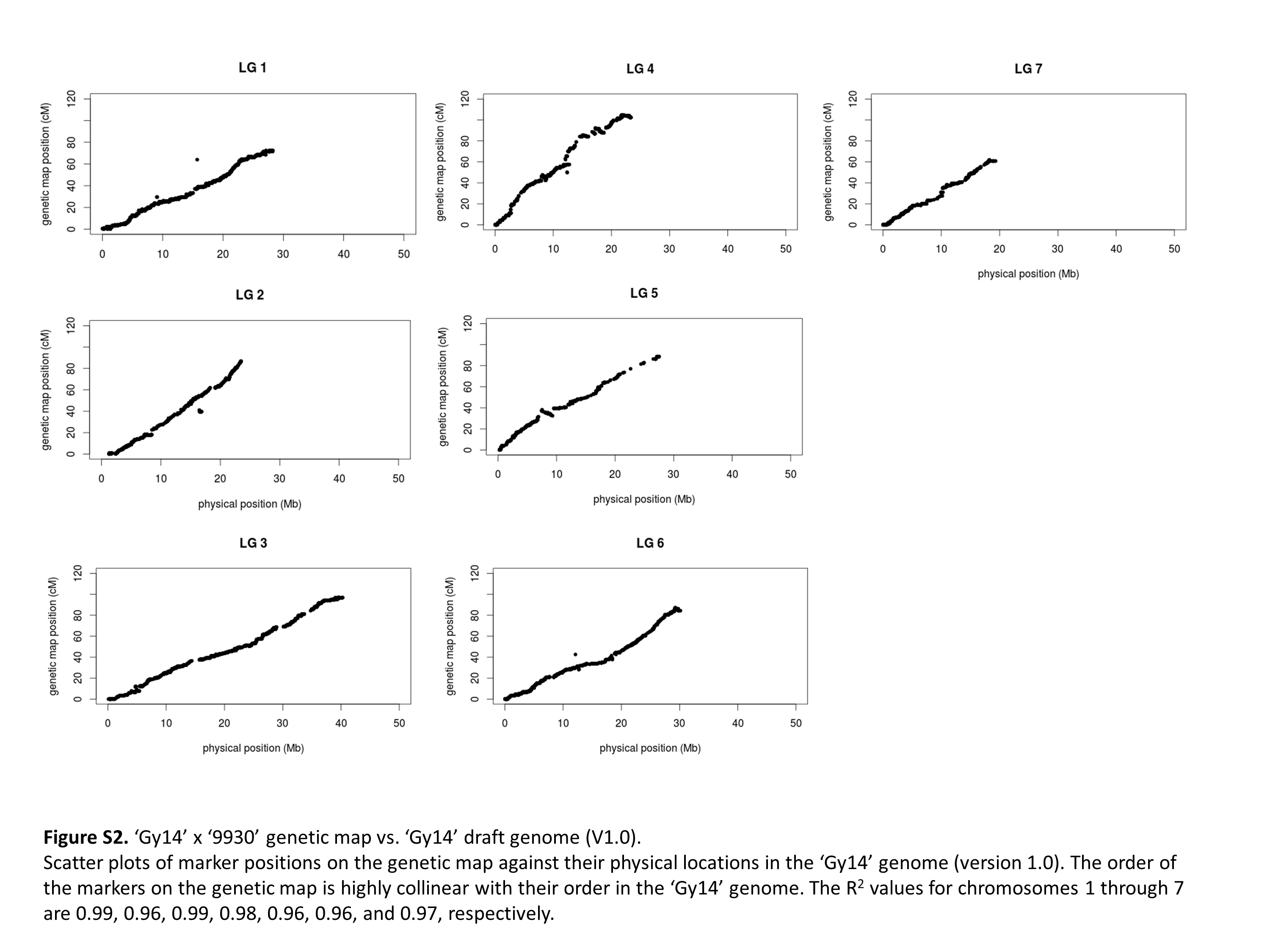

Supplement: S2 Fig — Scatter plots of marker positions on the genetic map against their physical locations in the ‘Gy14’ genome (version 1.0). The order of the markers on the genetic map is highly collinear with their order in the ‘Gy14’ genome. The R2 values for chromosomes 1 through 7 are 0.99, 0.96, 0.99, 0.98, 0.96, 0.96, and 0.97, respectively. (TIF) [file pone.0124101.s002.tif]

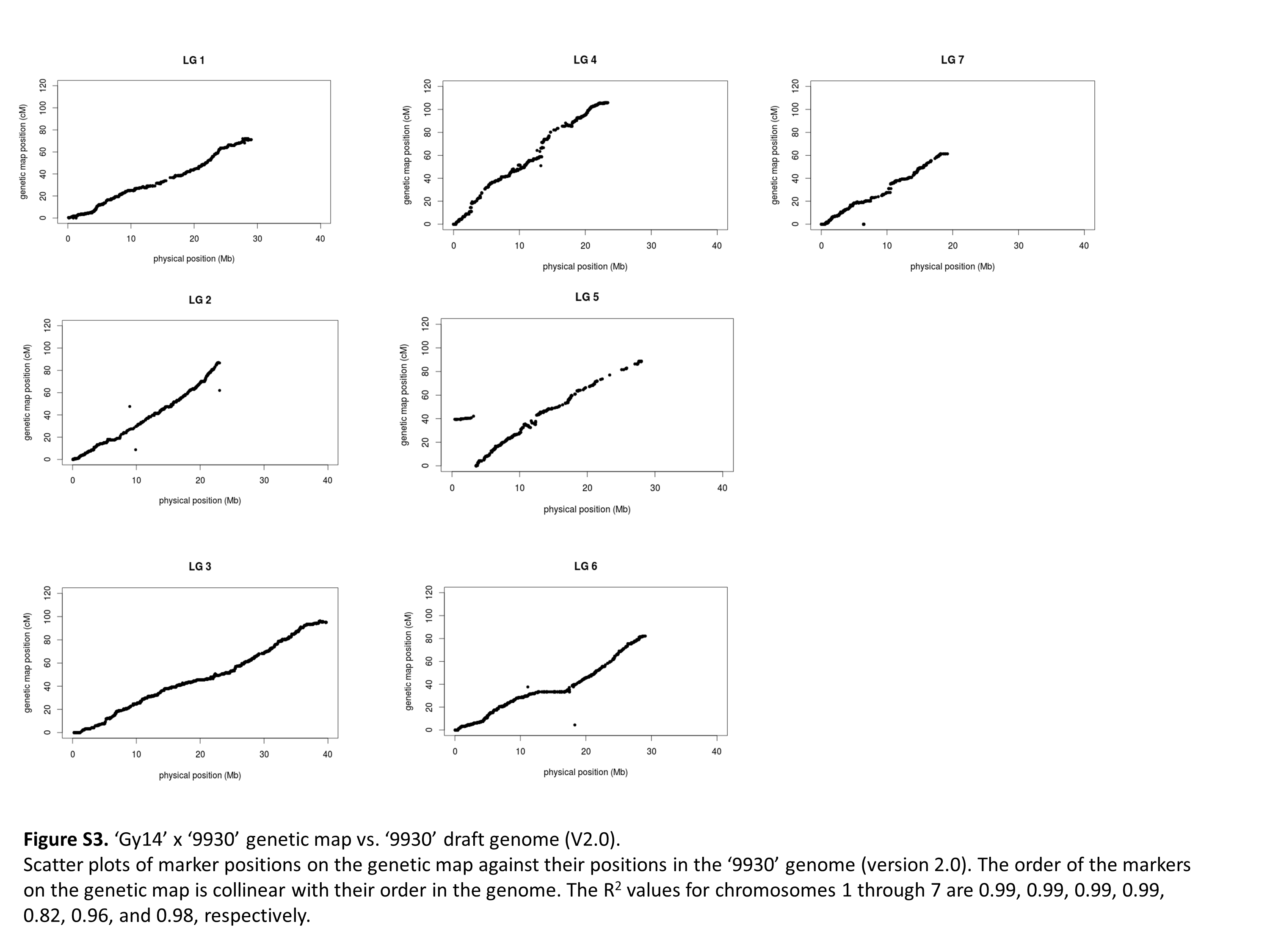

Supplement: S3 Fig — Scatter plots of marker positions on the genetic map against their positions in the ‘9930’ genome (version 2.0). The order of the markers on the genetic map is collinear with their order in the genome. The R2 values for chromosomes 1 through 7 are 0.99, 0.99, 0.99, 0.99, 0.82, 0.96, and 0.98, respectively. (TIF) [file pone.0124101.s003.tif]

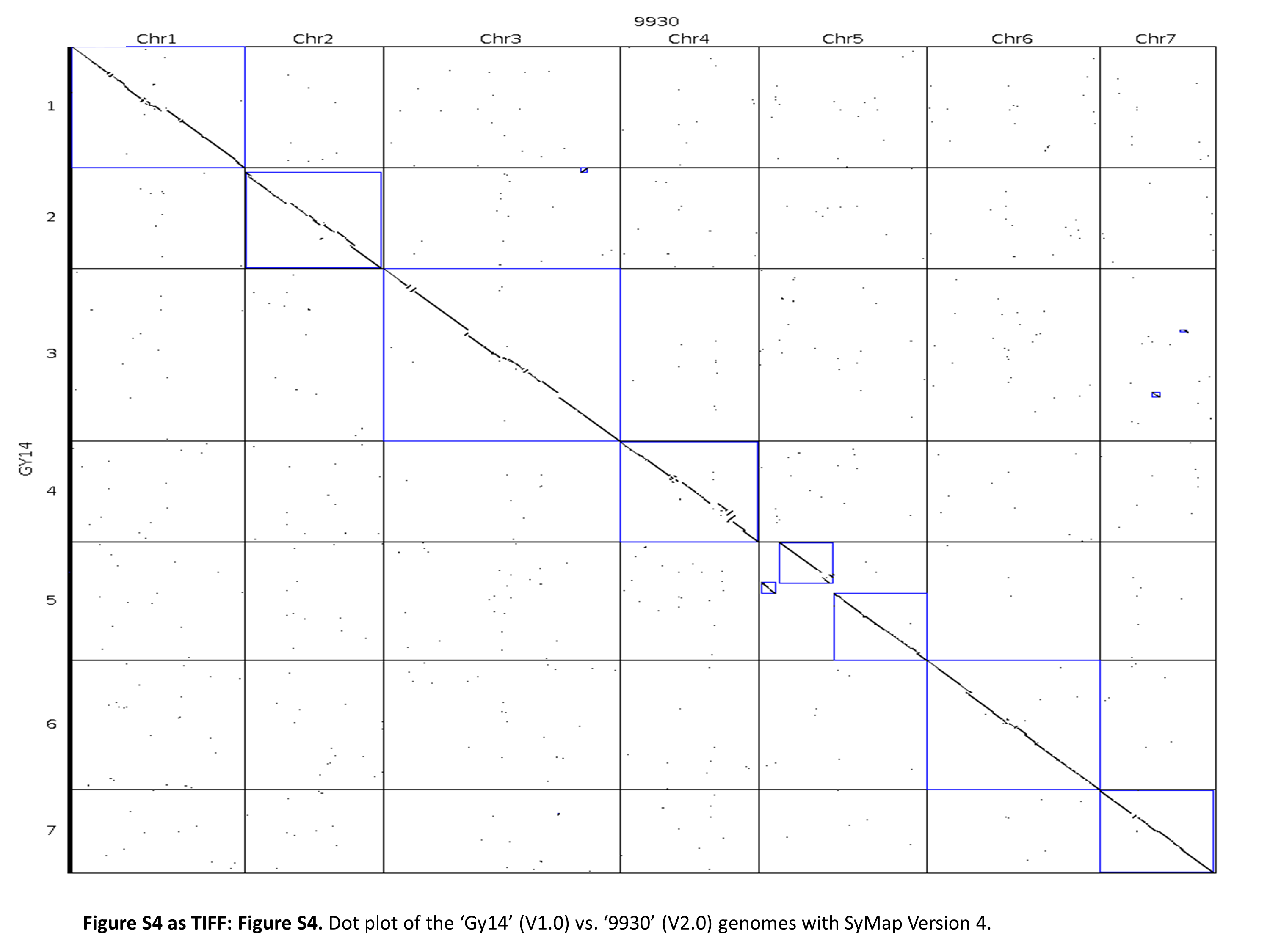

Supplement: S4 Fig — (TIF) [file pone.0124101.s004.tif]
